# Comprehensive and efficient analyses of T cell receptors for cancer antigens in peptide-vaccinated patients using a single cell-based gene cloning system

**DOI:** 10.1186/2051-1426-2-S3-P137

**Published:** 2014-11-06

**Authors:** Hidetoshi Nakagawa, Eishiro Mizukoshi, Eiji Kobayashi, Hiroyuki Kishi, Atsushi Muraguchi, Shuichi Kaneko

**Affiliations:** 1 Department of Disease control and Homeostasis, Kanazawa University, Kanazawa, Japan; 2 Department of Immunology, University of Toyama, Toyama, Japan

## Introduction

Obtaining T cell receptor repertoires specific for cancer antigens in individuals is of great significance in terms of their relationships with the clinical courses and therapeutic application such as adoptive transfer. However, full analyses of T cell receptor (TCR) sequences have been difficult because of the inefficiency of T cell cloning, heterogeneity of T cells or the heterodimeric structures of TCRs. Here, we revealed complete landscapes of cancer antigen-specific TCRs using a novel TCR cloning system; hTEC10 (**h**uman **T**CR **e**fficient **c**loning within **10 **days) [1].

## Methods

Fifteen patients were chosen from the participants of the Phase I clinical trial using HLA-A*2402-restricted α-fetoprotein (AFP)-derived peptide vaccine for advanced hepatocellular carcinoma (HCC). The peptide-specific cytotoxic lymphocytes (CTLs) were detected and sorted as single cells from the patients' peripheral mononuclear cells (PBMCs) after in vitro stimulation. cDNAs of paired TCR chains were amplified from the single cells, cloned into retroviral vectors, and transduced in the T cell strain or PBMCs. The avidities of the obtained TCRs were evaluated by comparing antigen-specific cytotoxicities. This system was also applied to the healthy controls and the participants of the Phase I clinical trial of human telomerase reverse transcriptase (hTERT)-derived peptide vaccine for prevention of HCC recurrence after radiofrequency ablation (RFA) therapy.

## Results and discussion

AFP-specific CTLs were induced in 4 patients whose clinical responses were CR (no recurrence on day 1,336), long SD (TTP, 812 days), SD (91 days) and PD (46 days), respectively (long SD is defined as SD over 6 months). Totally, 347 specific TCR clones that consisted of 10 kinds of TCR gene rearrangements (3 from the CR patient; 4 from the long SD patient; 2 from the SD patient and 1 from the PD patient) were amplified. The TCRs obtained from the CR patient and the long SD patient possessed higher avidities than that from the SD patient, the PD patient and the healthy controls without HCC. Similarly, we could obtain highly reactive 122 TCR clones consist of 10 different gene arrangements from 3 patients who were treated with hTERT vaccine and prevented from the recurrence until 24 weeks after RFA. These results indicated that the inductions of TCRs with high avidities reflected the good clinical responses, and high-avidity TCR might be retrieved from vaccinated patients with good clinical responses, not from patients with poor responses or healthy individuals.
